# Diagnostic Accuracy of NT-ProBNP for Heart Failure with Sepsis in Patients Younger than 18 Years

**DOI:** 10.1371/journal.pone.0147930

**Published:** 2016-01-26

**Authors:** Chun-wang Lin, Wen Tang, Fang Wen, Jin-jin Chen, Xiang-lin Zeng, Zong-geng Chen

**Affiliations:** 1 Affiliated Shunde Women and Children’s Hospital of Jinan University, Shunde, Guangdong, 528300, P. R. China; 2 First Affiliated Hospital of Zhongshan University, Guangzhou, Guangdong, 510632, P. R. China; Merck & Co., UNITED STATES

## Abstract

This clinical study investigated plasma NT-proBNP levels as a potential predictor of heart failure in pediatric patients with sepsis. Plasma NT-ProBNP levels of 211 pediatric patients with sepsis and 126 healthy children were measured. Patients were stratified as with heart failure (HF) or without heart failure (non-HF). Patients were graded as having sepsis, severe sepsis, or septic shock. The optimal cut-off values of plasma NT-ProBNP for heart failure were determined by analyzing the receiver operating characteristic (ROC). In the HF, non-HF and control groups, the median plasma NT-proBNP levels were 3640, 656, and 226 ng/L, respectively. For all patients with sepsis, the optimal diagnostic cut-off value was 1268 ng/L for differentiating heart failure. In the severe sepsis patients and septic shock patients, the optimal diagnostic cut-off values were 1368 ng/L and 1525 ng/L, respectively. This report is the first one to reveal that NT-proBNP may predict heart failure in children with sepsis. It provides an important clinical reference for the diagnosis of heart failure in pediatric patients with sepsis, and enables monitoring septic children for cardiac involvement.

## Introduction

Plasma NT-proBNP levels are often used as a biological marker of heart failure [1–8]. Heart failure may be diagnosed in adults younger than 50 years when NT-proBNP levels are >450 ng/L [9], and in children up to 14 years if ≥500 ng/L [10]. Higher-than-normal NT-proBNP levels are also a sign of systolic and diastolic dysfunction [11–14]. However, although the association between sepsis and cardiac dysfunction is well established [15], the response of plasma NT-proBNP to a septic condition has not been clearly demonstrated. Furthermore, patients with sepsis often have cardiovascular or other organ dysfunction, but a specific correlation between heart failure and NT-ProBNP in these patients is not clear. Therefore, when sepsis is present it is difficult to determine the relevancy of NT-proBNP for diagnosing heart failure.

This study delineated the association between plasma NT-proBNP levels and sepsis in children, and determined the appropriate cut-off values of plasma NT-proBNP for predicting heart failure in pediatric patients with sepsis.

## Materials and Methods

The Ethics Committee of Affiliated Shunde Women and Children’s Hospital of Jinan University and First Affiliated Hospital of Zhongshan University approved the study. All participants provided their written informed consent before the start of the research. We also obtained the written informed consent from the next of kin, caretakers, or guardians on behalf of the children enrolled in our study.

### Subjects

All subjects were recruited between June 2010 and June 2014 from Affiliated Shunde Women and Children’s Hospital of Jinan University and First Affiliated Hospital of Zhongshan University. Sepsis was defined by the International Consensus Conference on Pediatric Sepsis (2005) [16]. Heart failure was rated by the modified Ross score. In detail, the modified Ross scores for heart failure were: 0–2, none; 3–6, mild; 7–9, moderate; and 10–12, severe (Table 1) [17, 18]. Sepsis and heart failure were diagnosed by clinicians and confirmed by their senior colleagues without reference to NT-proBNP data.

**Table 1:** Modified Ross Score.

		0	+1	+2
Diaphoresis		Head only	Head & body ^a^	Head & body ^b^
Tachypnea		Rare	Several times	Frequent
Physical examination	Breathing	Normal	Retractions	Dyspnea
Respiratory rate, breathes/min	0–1 y	<50	50–60	>60
	1–6 y	<35	35–45	>45
	7–10 y	<25	25–35	>35
	11–14 y	<18	18–28	>28
Heart rate, beats/min	0–1 y	<160	160–170	>170
	1–6 y	<105	105–115	>115
	7–10 y	<90	90–100	>100
	11–14 y	<80	80–90	>90
Hepatomegaly size, cm		<2	2–3	>3

Total score: 0–2 (no HF), 3–6 (mild HF), 7–9 (moderate HF), 10–12 (severe HF)

^a^ At exertion

^b^ at rest

We consecutively enrolled 211 pediatric patients with sepsis. In complex congenital heart disease, the natriuretic peptide cut-offs for heart failure could differ from that of patients with structurally normal hearts, and therefore patients with complex congenital heart disease were excluded. Also excluded were patients with Kawasaki disease, nephropathy syndrome, nephritis, coronary heart disease, genetic cardiomyopathy, or pulmonary arterial hypertension disease, because these conditions affect plasma NT-proBNP levels. A control group of 126 healthy children were recruited.

Sepsis patients were divided into those with heart failure (HF group, n = 66) and those without heart failure (non-HF group, n = 145). Patients were also categorized as having sepsis, severe sepsis, or septic shock.

### Laboratory assays

The plasma NT-proBNP levels were measured and interpreted in a blind manner. Measurement of the NT-proBNP levels was fully automated to eliminate human error. A 2-mL venous blood sample was collected in a tube containing EDTA-K2. Blood samples from the sepsis patients were drawn on admission to the hospital; samples from the control group were drawn during routine check-ups.

The plasma NT-ProBNP concentration was measured by a RAMP fluorescence quantitative analyzer (Model YZB/CAN 91001, Canada Response Biomedical, Burnaby, Canada). The serum C reaction protein (CRP) concentration was measured using the turbidity method, and procalcitonin (PCT) was measured by using an automatic biochemical analyzer (Roche Diagnostics, model p800, Basel, Switzerland). Tumor necrosis factor alpha (TNF-α) and interleukin 6 (IL6) concentrations were measured with a chemical luminescence immunity analyzer (Siemens Medical Diagnostic Products, model Lknf1, Berlin, Germany).

Heart failure was diagnosed if the patient had a Ross score between 3 and 12, ejection fraction was < 50% (systolic heart), or E peak/A peak was < 1 (diastolic heart failure). The cut-off value of NT-ProBNP for heart failure diagnosis was estimated from the receiver operating characteristic (ROC) curve for plasma NT-ProBNP.

### Statistical analyses

The statistical analyses were performed with SPSS version 17.0 statistical software (SPSS, Chicago, IL, USA). Figures were generated using GraphPad software. (GraphPad Software, La Jolla, USA).

K-S testing was used to investigate the distribution of plasma NT-ProBNP levels in both the experimental and control groups. The results showed that distributions were not normal (Z = 2.701, *P* < 0.001; Z = 1.402, *P* = 0.041). The Kruskal Wallis test was used to compare the plasma NT-proBNP levels in patients of the sepsis HF, sepsis non-HF, and healthy control groups; and for the plasma NT-proBNP levels for patients with mild, moderate, and severe HF. Spearman’s test was applied for the correlation analysis between plasma NT-proBNP levels and heart rate, breath rate, liver enlargement, ejection fraction, and modified Ross score in all HF patients.

ROC curves were employed to determine the optimal cut-off values of plasma NT-ProBNP for heart failure in patients with sepsis, severe sepsis, or septic shock. The area under the ROC curve (AUC), sensitivity, specificity, positive and negative likelihood ratios, and 95% confidence intervals were calculated for the cutoff values. A probability of ≤ 0.05 was taken as significant.

## Results

The ages and genders of the sepsis (n = 211) and control (n = 126) groups were statistically similar (Table 1). Among the 211 sepsis patients, 85, 74, and 52 were determined to have sepsis, severe sepsis, and septic shock, respectively. Of the 211 patients, 66 had HF (31.3%), and 145 (68.7%) were in the non-HF group. Of the 66 HF patients, 6 (7.1%) had sepsis, 25 (33.8%) had severe sepsis, and 35 (67.3%) had septic shock. All HF patients had left ventricle systolic dysfunction (systolic HF) with an ejection fraction < 50% (50–75% is considered normal), and no diastolic HF (ratios of the E-to-A peaks were >1 and < 3; 1–3 considered normal) in the heart Doppler examination [19, 20]. Gender- and age-matched healthy subjects (65 boys and 61 girls) had an average age of 4.1 years.

The primary symptoms of sepsis included severe lower respiratory tract infection (n = 89), meningoencephalitis (n = 29), viral myocarditis (n = 23), severe salmonella enteritidis (n = 9), severe pancreatitis (n = 9), toxic bacillary dysentery (n = 8), cellulitis (n = 8), severe entero virus 71 infection (n = 7), liver abscess (n = 6), infective endocarditis (n = 5), postoperative appendicitis (n = 5), cholangitis (n = 4), severe burn complicated by infection (n = 3), severe trauma (n = 2), lung abscess (n = 2), and urinary tract infection (n = 2).

**Fig 1:**
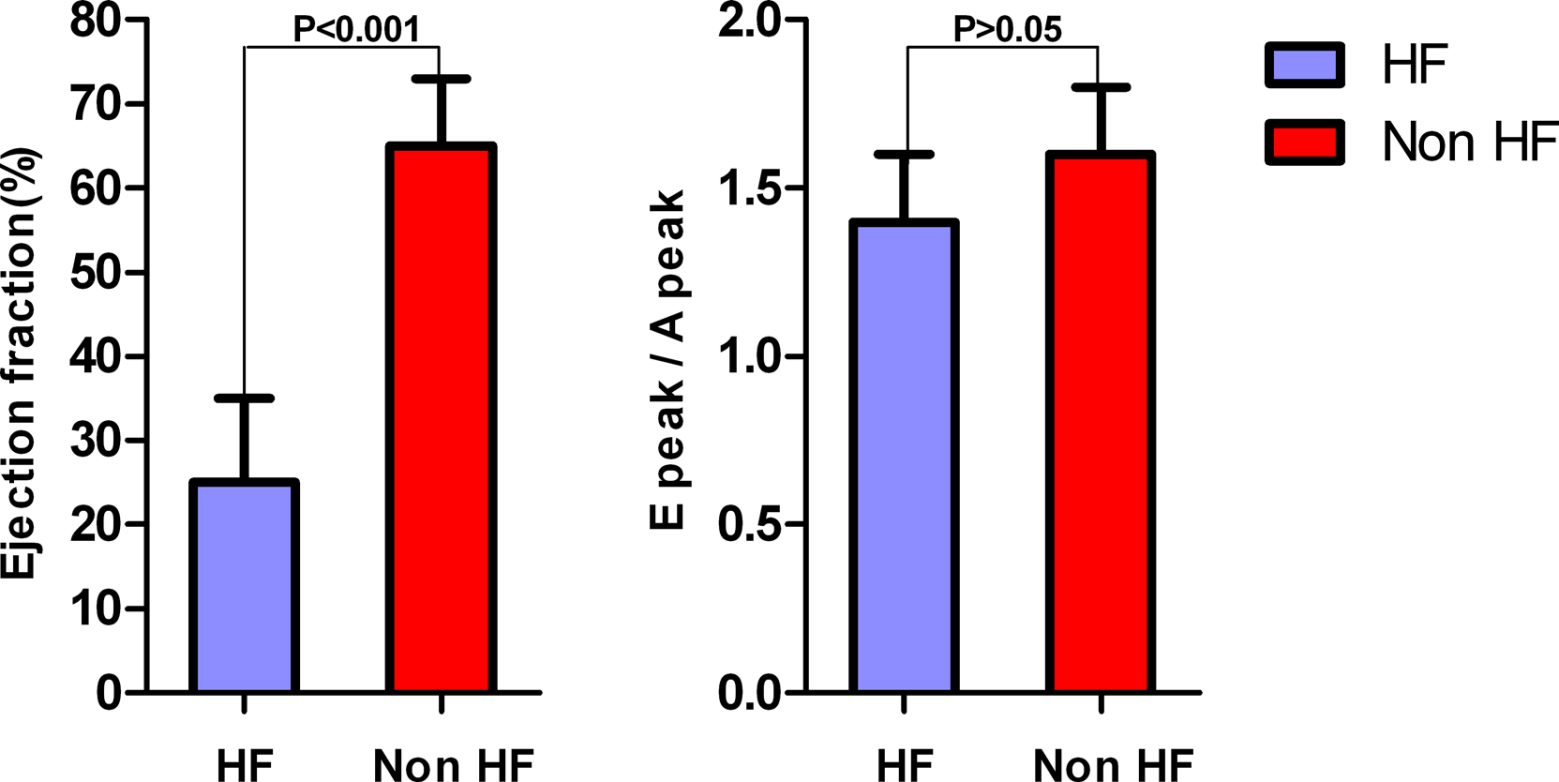
In the heart Doppler examination, the ejection fractions in HF and non-HF patients with sepsis were 25% ± 10.4% and 65% ± 7.5%, respectively, a significant difference (*P* < 0.001); the E peak/A peak of the 2 groups were normal and not significantly different (1.4 ± 0.2 cf. 1.6 ± 0.2). The results suggested a left ventricle systolic dysfunction in all heart failure patients with sepsis.

The ultrasonic cardiogram results at first administration are shown in Table 2. The ejection fractions in HF and non-HF patients were 25% ± 10.4% and 65% ± 7.5%, respectively, a significant difference (*P* < 0.001). The E peak/A peak of the 2 groups were normal and not significantly different (1.4 ± 0.2 cf. 1.6 ± 0.2). The results suggested a left ventricle systolic dysfunction in all HF patients (Fig 1).

**Table 2:** Characteristics of pediatric sepsis patients at administration.

	All patients	HF	Non-HF	*P*
Number of subjects	211	66	145	—
Age, y	3.6 ± 1.3	3.6 ± 1.5	3.6 ± 1.2	0.25
Male %	51.5	51.3	51.7	0.86
NT-proBNP, ng/L	1396	3640	656	<0.001
Body temperature, °C	39 ± 0.7	39 ± 0.8	39 ± 0.5	0.58
Heart rate, beats/min	120 ± 15	145 ± 20	95 ± 10	<0.05
Breath rate, breathes/min	45 ± 5	55 ± 6	35 ± 4	<0.05
Liver enlargement, cm	2 ± 1.0	4 ± 0.5	1 ± 0.5	<0.001
Plasma glucose, mmol/L	8.7 ± 1.8	9.6 ± 1.7	7.8 ± 1.8	0.17
White blood cell increase, ×10^9^/ L	15 ± 2.7	16 ± 3.1	14 ± 2.3	0.47
White blood cell decrease, ×10^9^/ L	3.0 ± 0.8	3.0 ± 0.7	3.0 ± 0.9	0.28
Platelet, 10 × 10^9^/ L	7.4 ± 0.3	5.3 ± 0.2	9.4 ± 0.4	<0.05
Hemoglobin, g/L	69 ± 14	62 ± 12	87 ± 17	0.07
Serum total bilirubin, mmol/L	77 ± 23.3	98 ± 33.8	55 ± 12.7	<0.05
Alanine transaminase, U/L	59 ± 17	82 ± 15	36 ± 18	<0.001
C reaction protein, mg/L	39 ± 6.4	48 ± 11.2	29 ± 0.6	<0.05
Procalcitonin, mg/L	14 ± 1.1	16 ± 2.6	11 ± 0.5	<0.001
Blood lactic acid, mmol/L	5 ± 0.9	6 ± 1.2	4 ± 0.6	<0.05
Activated partial thromboplastin time, s	70 ± 14.8	95 ± 12.5	45 ± 17.1	<0.001
TNF-α, pg/mL	42 ± 15.2	52 ± 15.3	31 ± 16.1	<0.05
IL6, pg/mL	61 ± 11.3	77 ± 11.9	44 ± 10.6	<0.05
Ejection fraction, %	45 ± 9.5	25 ± 10.4	65 ± 8.5	<0.001
E peak /A peak	1.5± 0.2	1.4± 0.2	1.6± 0.2	0.12
Arterial oxygen saturation, %	44 ± 14.9	33 ± 16.4	55 ± 13.4	<0.05
Systolic blood pressure, mmHg	89 ± 11.5	85 ± 11.6	92 ± 12.5	0.42
Urine volume, mg·kg^-1^·min^-1^	0.3 ± 0.05	0.2 ± 0.05	0.4 ± 0.05	<0.05
Capillary filling, s	4 ± 1.0	5 ± 1.0	3 ± 1.0	<0.05

The severity of heart failure was evaluated on the basis of ejection fraction and results showed that there were 14 (21.2%), 19 (28.8%), and 33 (50%) patients with mild, moderate, and severe heart failure, respectively. Patients with moderate or severe heart failure were among the patients with severe sepsis or septic shock.

The plasma NT-proBNP levels were not normally distributed (*P* < 0.001) and therefore the median was used. There was a positive correlation between plasma NT-proBNP levels and heart rate (*r* = 0.754, *P* < 0.001), breath rate (*r* = 0.659, *P* < 0.001), and liver enlargement (*r* = 0.651, *P* < 0.001). NT-proBNP levels negatively correlated with ejection fraction in these sepsis patients (*r* = –0.782, *P* < 0.001); the F-test for linear regression reached statistical significance (*P* < 0.001; Table 3).

**Table 3:** The correlation between plasma NT-proBNP level and heart rate, breathe rate, liver enlargement, and Ejection fraction in the all heart failure patients.

Related factors	r	P	F	R
Heart rate, beats/min	0.754	0.000	77.65	0.573
Breath rate, breathes/min	0.659	0.000	56.86	0.494
Liver enlargement, cm	0.651	0.000	56.36	0.486
Ejection fraction, %	–0.782	0.000	78.87	0.588

In the 66 septic patients with HF, the median plasma NT-proBNP levels of mild, moderate, and severe HF were 1348.22 ng/L (n = 32), 3472.15 ng/L (n = 19), and 4236.23 ng/L (n = 11), respectively; the differences among them were significant (*x*^2^ = 41.13, *P* < 0.001). The plasma NT-proBNP level rises with the increasing modified Ross score; there was a positive correlation between NT-proBNP level and modified Ross score (*r* = 0.679, *P* < 0.001).

**Fig 2:**
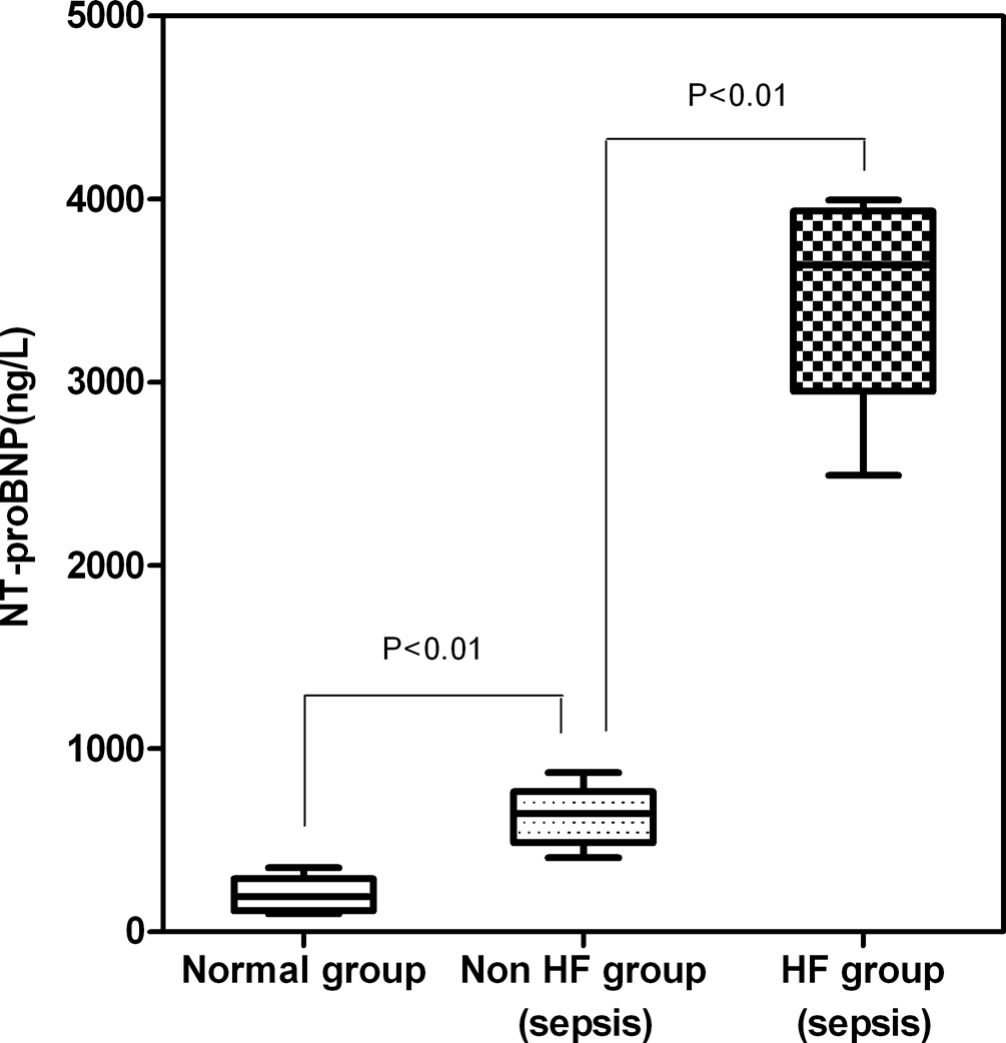
The median plasma NT-proBNP levels of the sepsis HF, sepsis non-HF, and healthy control groups were 3885 ng/L, 656 ng/Land 226 ng/L, respectively; the differences among them were significant (χ^2^ = 41.87, *P* < 0.001).

The median plasma NT-proBNP levels of the sepsis HF, sepsis non-HF, and healthy control groups were 3885 ng/L, 656 ng/Land 226 ng/L, respectively; the differences among them were significant (χ^2^ = 41.87, *P* < 0.001; Fig 2). Among the sepsis HF patients, the median plasma NT-proBNP levels of those with severe sepsis and those with septic shock were 4565 ng/L and 5872 ng/L, respectively.

The median plasma NT-proBNP levels were 656 ng/L (465–2150 ng/L) in non-HF sepsis patients, 1350 ng/L (1200–3054 ng/L) in non-HF severe sepsis patients, and 1425 ng/L (1250–3225 ng/L) in non-HF septic shock patients. The ejection fraction and E peak/A peak were normal in all non-HF sepsis patients.

Among the 211 patients, 187 recovered (average hospitalization was 12.5 days) and 24 died (average hospitalization was 5.5 days), including 10 with severe sepsis (4.74%) and 14 with septic shock (6.64%). Plasma NT-proBNP levels of the surviving and deceased patients were not significantly different (4486 ng/L cf. 4618 ng/L in patients with severe sepsis; 4774 ng/L cf. 4951 ng/ L in patients with septic shock).

Among the 66 septic patients with HF, 17 patients died. In the 49 surviving patients, the average recovery time for HF and sepsis were 1.5 days and 14.5 days, respectively; the median plasma NT-proBNP levels were 3000.15 ng/L before treatment, 1685.22 ng/L after recovery of HF, and 329.35 ng/L after recovery of sepsis, which showed the potential effects of sepsis on NT- proBNP; the differences among them and between any two comparisons (before treatment and after HF recovery, before treatment and after sepsis recovery, and after HF and sepsis recovery) were significant (*P* < 0.001 for all; Table 4).

**Table 4:** Comparison of plasma NT-proBNP levels before treament and after HF and sepsis recovery, ng/L.

	NT-proBNP	*Z*-value	*P*-value
Before treatment	3000.15	–3.862	0.000
After HF recovered	1685.22		
Before treatment	3000.15	–5.238	0.000
After sepsis recovered	329.35		
After HF recovered	1685.22	–4.159	0.000
After sepsis recovered	329.35		

In 187 surviving patients, the median plasma NT-proBNP level was 986 ng/L before treatment and significantly lower (312 ng/L) after treatment (*P* < 0.001).

Sicker patients with severe sepsis (n = 74) and with septic shock (n = 52) required more fluid for treatment. Patients were stratified as group A or group B, according to the total per capita infusion in the first week, as follows. Patients with severe sepsis were considered in the A1 or B1 group if they received ≤6000 mL or >6000 mL infusions, respectively. Patients with septic shock were in the A2 or B2 group if they received ≤9000 mL or >9000 mL. In the A1 and B1 groups, NT-proBNP levels were 2056 ng/L and 2462 ng/L, respectively (*P =* 0.09). In the A2 and B2 groups, NT-proBNP levels were 3168 ng/L and 3719 ng/L (*P* = 0.08). There was no significant difference between patients receiving high volume and lower volume transfusions.

Among the sepsis patients, plasma NT-proBNP levels positively correlated with CRP (*r* = 0.544), procalcitonin (*r* = 0.815), TNF-α (*r* = 0.712), and IL6 (*r* = 0.682; *P* < 0.001 for all).

**Fig 3:**
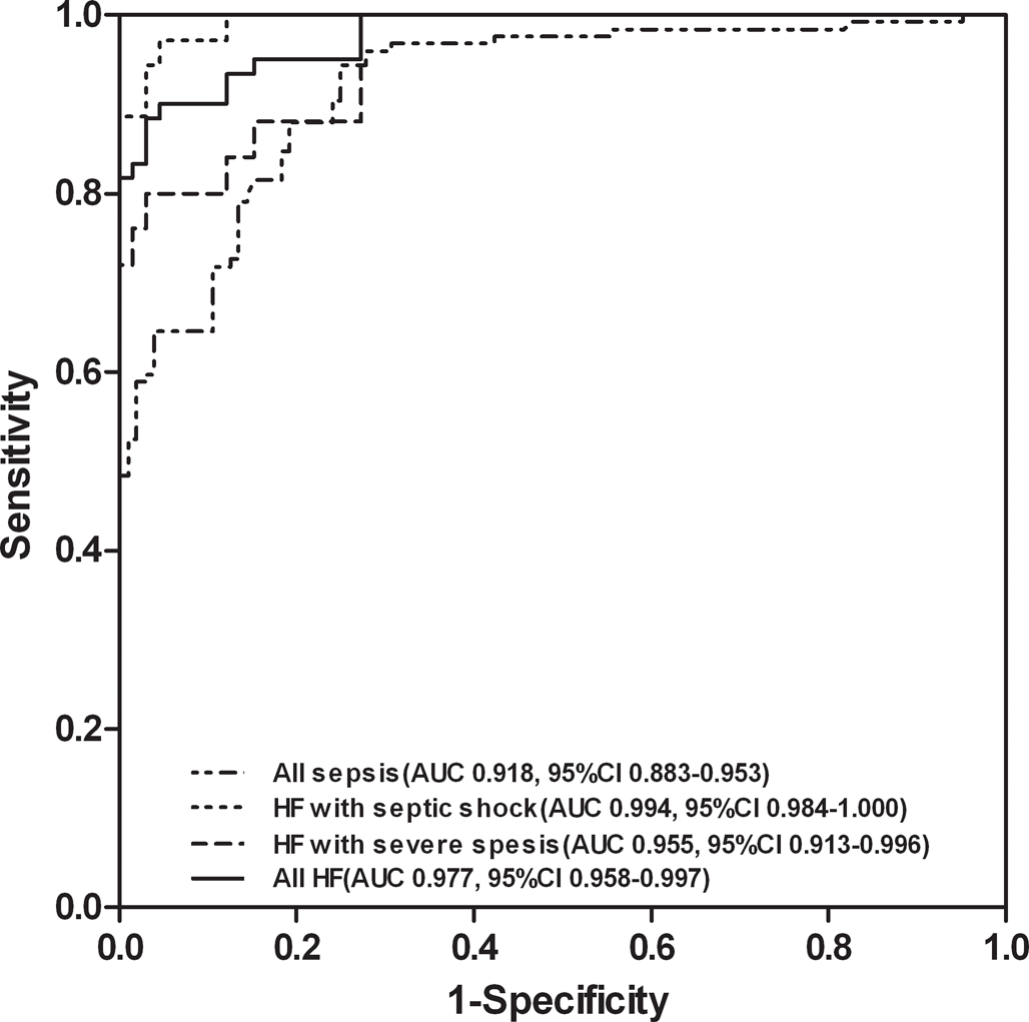
Comparing ROC curves for plasma NT-proBNP in all pediatric patients with sepsis, HF patients with severe sepsis, HF patients with septic shock, and all HF patients with sepsis.

To estimate the diagnostic accuracy of plasma NT-proBNP levels to detect heart failure in all sepsis patients, severe sepsis patients and septic shock patients, ROC curve analysis was employed (Fig 3; Table 5). The optimal diagnostic cut-off value for heart failure was 1268 ng/L (positive likelihood ratio = 11.9) sepsis patients, 1368 ng/L (positive likelihood ratio = 13.2) in severe sepsis patients, and 1525 ng/L (positive likelihood ratio = 21.4) in patients with septic shock (Table 5). The diagnostic sensitivity and specificity were shown in Table 5.

**Table 5:** ROC analysis determining viability of plasma NT-ProBNP levels for differentiating heart failure in sepsis patients.*

	All	Severe sepsis	Septic shock
Subjects, n	211	74	52
Optimal cut-off, ng/L	1268	1368	1525
AUC	0.977 (0.958–0.997)	0.955 (0.913–0.996)	0.994 (0.984–1.000)
Sensitivity	0.900 (0.795–0.962)	0.800 (0.593–0.932)	0.971 (0.851–0.999)
Specificity	0.924 (0.832–0.975)	0.939 (0.852–0.983)	0.955 (0.873–0.991)
Positive likelihood ratio	11.9	13.2	21.4

* Reported as value (95% CI), unless otherwise noted

## Discussion

This study investigated whether plasma NT-proBNP levels can be used to predict heart failure in pediatric patients with sepsis. The plasma NT-proBNP levels of pediatric patients with sepsis (including patients with heart failure) were evaluated and compared with age- and gender-matched healthy children. Using plasma NT-proBNP as an index for the diagnosis of heart failure has been accepted and widely used; the commonly used diagnostic cut-off points (>450 ng/L for adults and >500 ng/L for children), however, may not be appropriate for septic patients. This study found that NT-proBNP levels in sepsis patients were significantly higher than that of non-sepsis patients, suggesting the need to determine the appropriate diagnostic cut-off points for heart failure in septic patients that will be conducive to the management of septic patients with heart failure. We found that septic patients with heart failure had a left ventricle systolic dysfunction and plasma NT-ProBNP levels were significantly elevated in septic patients (with or without heart failure), relative to the healthy control group. NT-ProBNP levels were also significantly higher in patients with heart failure compared with those without heart failure. The optimal cut-off value of NT-ProBNP for heart failure in all patients was 1268 ng/L, and the diagnostic sensitivity, specificity, and accuracy were 90.0%, 92.4%, and 0.912%, respectively, with a positive likelihood ratio of 11.9. These results support plasma NT-ProBNP as a valid predictor of heart failure in children with sepsis.

Myocardial dysfunction is a common complication in septic patients. Caksen et al. [21] observed pericardial effusion in 20% of pediatric patients with sepsis caused by *Staphylococcus aureus*. In this study, the median plasma NT-proBNP level of sepsis patients without heart failure (656 ng/L) was significantly higher than that of healthy children (226 ng/L). This is also higher than the 500 ng/L for diagnosing heart failure in general pediatric patients [10]. In the 49 surviving patients with heart failure, the plasma NT-proBNP levels were significantly reduced but still as high as 1685.22 ng/L after heart failure recovery. However, plasma NT-proBNP levels decreased to normal (329.35 ng/L) after sepsis recovery, which showed the potential effect of sepsis on NT-proBNP levels.

This study suggests that sepsis may contribute to heart failure. These findings are consistent with those of previous reports that NT-proBNP strongly correlates with systolic dysfunction and diastolic dysfunction in patients with severe sepsis or septic shock. Other studies have shown that high NT-proBNP was associated with mortality in adult patients with severe sepsis [22], or in pediatric patients with severe sepsis or septic shock [23]. A meta-analysis further showed that an elevated NT-proBNP is a strong predictor of high mortality in septic patients [24].

This study showed that sicker patients with sepsis required more fluid for treatment, which did not increase NT-proBNP levels significantly (*P* > 0.05), and affect NT-proBNP for diagnosis of septic heart failure.

This study shows that NT-proBNP is associated with the severity of sepsis but is not a predictor of death in patients with severe sepsis or septic shock.

The cardiovascular response to septic shock is peripheral vasodilatation, resulting from myocardial depression and ventricular dilation, possibly caused by endotoxins from Gram-negative bacteria [25]. In our study, NT-proBNP levels positively correlated with the inflammatory molecules CRP, procalcitonin, TNF-α and IL6, suggesting that the inflammatory response may contribute to cardiac dysfunctions and indirectly elevates circulatory NT-proBNP levels. It is noteworthy that a previous study showed that in vitro exposure to TNF-α or IL6 isolated from serum of humans with septic shock induced depression of rat cardiac myocyte contractile function, and these two factors appeared to have a synergistic effect [26].

In the present study, the cut-off value of NT-proBNP for heart failure in patients with septic shock (1525 ng/L) was significantly higher than that of patients with severe sepsis (1368 ng/L), suggesting that septic shock has a greater influence on NT-proBNP levels than sepsis does. This could be due to inflammatory factors released during septic shock. Further investigation is warranted to elucidate the mechanism underlying the correlation between plasma NT-proBNP and measured inflammatory factors.

## Conclusion

This report is the first one to reveal that NT-proBNP may predict heart failure in children with sepsis. In addition, it provides an important clinical reference for the diagnosis of heart failure in pediatric patients with sepsis, and enables monitoring septic children for cardiac involvement.
